# Cisplatin‐induced HSF1‐HSP90 axis enhances the expression of functional PD‐L1 in oral squamous cell carcinoma

**DOI:** 10.1002/cam4.5310

**Published:** 2022-10-06

**Authors:** Takashi Sasaya, Terufumi Kubo, Kenji Murata, Yuka Mizue, Kenta Sasaki, Junko Yanagawa, Makoto Imagawa, Hirotaka Kato, Tomohide Tsukahara, Takayuki Kanaseki, Yasuaki Tamura, Akihiro Miyazaki, Yoshihiko Hirohashi, Toshihiko Torigoe

**Affiliations:** ^1^ Department of Pathology Sapporo Medical University School of Medicine Sapporo Japan; ^2^ Department of Oral Surgery Sapporo Medical University School of Medicine Sapporo Japan

**Keywords:** cisplatin, heat shock factor 1, heat shock protein 90, oral squamous cell carcinoma, PD‐L1

## Abstract

Immune checkpoint inhibitor‐based cancer immunotherapy has provided an additional therapeutic option for oral squamous cell carcinoma (OSCC) with recurrence or distant metastases. However, further improvement of OSCC treatment is required to develop the optimal combination or order for chemoradiotherapy and immunotherapy. Along with the accumulation of clinical knowledge and evidence, it is also essential to clarify the biological impact of chemo‐radiotherapeutic agents on the cancer immune microenvironment. In this study, we investigated the effects of cisplatin (CDDP), a key therapeutic agent for OSCC, on programmed death‐ligand 1 (PD‐L1) expression in OSCC lines. Although CDDP treatment increased the surface levels of PD‐L1 on OSCC cell lines, the gene and total protein expression levels of PD‐L1 were not altered. We also demonstrated that the phosphorylation of heat shock factor 1 and heat shock protein 90 was involved in this process. In addition, CDDP‐induced PD‐L1 attenuated the target‐specific cytotoxic T lymphocyte reaction to OSCC. These results provide an immunobiological basis for the response of OSCC to CDDP and will contribute to our biological understanding of the action of novel combination therapy including immunotherapy together with platinum‐based chemotherapy for OSCC.

## Abstract 2

In this report, we demonstrated that CDDP increased the cell surface expression of PD‐L1 on HSC2 OSCC cells and was mediated by HSF1 and HSP90.

## 1 INTRODUCTION

Head and neck cancer is the sixth most commonly diagnosed cancer worldwide, and oral cancer is the most frequent tumor of the head and neck region. More than 90% of oral cancer is classified as oral squamous cell carcinoma (OSCC), with 377,713 new cases and 177,757 deaths worldwide in 2020.^1^, ^2^ Together with surgery, chemotherapy in combination with radiotherapy for OSCC have been developed in the past couple of decades. Cisplatin (CDDP), the most classic platinum‐based cytotoxic agent adapted to various types of malignancies, remains a pivotally important chemotherapeutic drug for OSCC. Although intra‐arterial infusion chemotherapy including CDDP has shown excellent clinical outcomes, research on other nonsurgical approaches for OSCC is ongoing.^3^ Interestingly, other than the standard cancer prognostic factors such as tumor invasion depth or histological grade, lymphocyte infiltration, and the expression of human leukocyte antigen (HLA) class I molecules, the prerequisite factors in cancer immunity are profoundly involved in the survival rate of OSCC patients.^4^, ^5^ These observations indicate that immune surveillance for OSCC is functionally important in the regulation of OSCC. Indeed, since the beginning of this century, immune checkpoint inhibitor (ICI)‐based cancer immunotherapy has achieved great success in some types of malignancies, including OSCC. Squamous cell carcinoma, including OSCC, tends to have a high tumor mutational burden and is considered to be immunogenic and show a favorable response to ICI‐mediated cancer immunotherapy even with single ICI agents.^6^, ^7^, ^8^ Recent studies have shown that cytotoxic chemotherapeutics including CDDP participate in autologous immune reactions against malignant cells via two mechanisms.^9^, ^10^ In the context of enhancing cancer immunity, cytotoxic chemotherapeutics evoke immunogenic cell death or reduce the number of regulatory T lymphocytes. However, such drugs can also suppress immunity. Therefore, it has become increasingly important that clinical and basic studies investigate the combined effect of these drugs together with existing treatment approaches and cancer immunity.

The interaction between programmed death‐1 (PD‐1) on T cells and programmed death‐ligand 1 (PD‐L1) on malignant cells, the so‐called effector phase, plays a crucial role in the immune escape of cancer cells, as shown by the clinical efficacy of blocking PD‐1/PD‐L1 in current cancer immunotherapy.^11^ PD‐L1 expression is not solely dependent on activated cytotoxic T lymphocyte (CTL)‐derived interferon gamma (IFNγ) but is also regulated by numerous factors.^12^ Accumulating evidence has shown that non‐IFNγ stimulation, including chemotherapeutic agents, induces PD‐L1 expression on malignant cells, although the mechanism and its significance in the cancer microenvironment have not been clarified fully. Thus, it is important to understand and develop cancer immunotherapy, especially in relation to its combination with other cancer therapeutic modalities.

In this study, we investigated whether CDDP controls the immunogenicity of OSCC cells and found that CDDP increased the surface levels of PD‐L1 on OSCC cells through cell stress signaling.

## 2 MATERIALS AND METHODS

### 2.1 Cell culture and stimulation

The OSCC cell lines HSC2 (expressing HLA‐A24) and HSC4 were purchased from the Human Science Research Resources Bank (Osaka, Japan). These cell lines were maintained in Dulbecco's modified Eagle's medium (Nacalai Tesque, Kyoto, Japan) supplemented with 10% fetal bovine serum (Sigma‐Aldrich) and 1% penicillin–streptomycin (10,000 U/ml penicillin, 10,000 μg/ml streptomycin; Life Technologies). Platinum‐A retroviral packaging Cell (Cell Biolabs) were cultured in Dulbecco's modified Eagle's medium supplemented with 10% fetal bovine serum, 10 μg/ml blasticidin (Sigma‐Aldrich), and 1 μg/ml puromycin (Sigma‐Aldrich). The cells were cultured in a humidified 5% CO_2_ incubator at 37°C.

In the indicated experiments, the cells were incubated in culture medium with or without CDDP (Nichi‐Iko Pharmaceutical Co., Ltd.) or IFNγ (PeproTech). The heat shock protein (HSP) 90 inhibitors 17‐AAG (tanespimycin) and TAS‐116 (pimitespib) were purchased from Abcam (Cambridge, UK) and MedChemExpress (Monmouth Junction, NJ), respectively. The anti‐PD‐1 antibody nivolumab was provided by Ono Pharmaceutical (Osaka, Japan).

### 2.2 AKF9 neoantigen and AKF9‐targeting T cell receptor‐engineered T (TCR‐T) cells

AKF9 is an HLA‐A24 restricted neoantigen that was derived from the *AP2S1* gene mutation, which was originally identified in the HCT15 colon adenocarcinoma cell line. Previously, we established a CTL clone that is highly reactive to this neoantigen.^13^, ^14^ In the present study, we cloned the TCR of this CTL clone and transfected it into CD8^+^ T cells. The detailed method for establishing TCR‐T cells is described elsewhere.^15^ This AKF9 neoantigen‐specific TCR‐T cell line was maintained in AIM‐V (Life Technologies) supplemented with 10% human serum, 1% HEPES (Life Technologies), 1% penicillin–streptomycin (Life Technologies), 0.1% of 2‐mercaptoethanol (Life Technologies), and 100 U/ml interleukin‐2 (PeproTech).

### 2.3 Flow cytometric analysis

HSC2 or HSC4 cells were seeded at a density of 1.0 × 10^6^ cells in a 60‐mm dish, and upon reaching sub‐confluence, the cells were collected by trypsinization, washed with phosphate‐buffered saline, and stained with a PE‐labeled antihuman CD274 (PD‐L1) antibody (clone 29E.2A3; BioLegend) or CD273 (PD‐L2) antibody (clone MIH18; BioLegend) for 30 min. AKF9‐targeting CTLs or TCR‐T cells were stained with an FITC‐labeled antihuman CD279 (PD‐1) antibody (BioLegend) for 30 min. The labeled cells were obtained and analyzed using a FACSCalibur flow cytometer (BD) and FlowJo (Tree Star, Inc.), respectively. We calculated the relative difference of mean fluorescence intensity, which indicated the cell surface expression of PD‐L1 or PD‐L2. An isotype‐matched antibody was used for control staining.

### 2.4 HSP27 mRNA knockdown by small interfering RNA (siRNA)

HSC2 cells were transfected with siRNA targeting HSP27 or negative control siRNA (Ambion, Austin, TX). Human HSP27‐specific siRNAs were purchased from Ambion: siRNA #1 sense, 5′‐GCGUGUCCCUGGAUGUCAAtt‐3′ and antisense, 5′‐UUGACAUCCAGGGACACGCgc‐3′; siRNA #2 sense, 5′‐GCCGCCAAGUAAAGCCUUAtt‐3′ and antisense, 5′‐UAAGGCUUUACUUGGCGGCag‐3′. At 48 h after transfection, the cells were incubated in culture medium with or without 10 μM CDDP for an additional 24 h before analysis.

### 2.5 Western blot analysis

The protocol for western blot analysis has been described elsewhere.^16^ The following antibodies were used: rabbit anti‐PD‐L1 monoclonal antibody (mAb) (clone 28‐8; Abcam) rabbit anti‐HSP27 polyclonal antibody (ab5579; Abcam), mouse anti‐HSP70 mAb (clone C92F3A‐5; Abcam), rabbit anti‐HSP90 polyclonal antibody (#4874; Cell Signaling Technology, Danvers, MA), rabbit antiheat shock factor (HSF) 1 mAb (clone EP1710Y; Abcam), rabbit anti‐HSF1 phospho S326 mAb (clone EP1713Y; Abcam), rabbit anti‐HSF1 phospho S320 mAb (clone EPR1712; Abcam), and anti‐β‐actin mAb (clone AC‐15; Sigma‐Aldrich). Peroxidase‐conjugated goat antimouse and antirabbit IgGs were purchased from KPL.

### 2.6 Fluor‐immunohistochemical analysis

The protocol for fluor‐immunohistochemical analysis has been described previously.^17^ For PD‐L1 staining, a rabbit anti‐PD‐L1 mAb (clone 28‐8) was used. Specimens were examined under an immunofluorescence microscope (BZ‐X700).

### 2.7 Quantitative real‐time PCR analysis

HSC2 cells were seeded at a density of 3.0 × 10^5^ cells in 6‐well plates for quantitative real‐time PCR analysis. Total RNA was isolated from cultured cells using an RNeasy Mini Kit (Qiagen). Quantitative real‐time PCR was performed as described previously.^18^ HSF1, PD‐L1, and GAPDH probes were designed by Life Technologies (TaqMan Gene expression assay).

### 2.8 Immunoprecipitation

Immunoprecipitation was performed using an Immunoprecipitation Kit (Abcam). Briefly, cell lysates were incubated with PD‐L1 (E1L3N) XP Rabbit mAb (Cell Signaling Technology) or rabbit gamma globulin control (Invitrogen) at 4°C overnight on a rotary mixer. To precipitate the complexes, protein A/G‐Sepharose beads were added and incubated for 1 h at 4°C. After thorough washing with wash buffer, the beads were boiled in SDS‐PAGE loading buffer before western blot analysis.

### 2.9 IFNγ enzyme‐linked immunospot (ELISpot) assay

An IFNγ ELISpot assay was performed as previously described.^13^ Briefly, 1.0 × 10^5^ AKF9‐specific TCR‐T cells or CTL clone cells were incubated with 1.0 × 10^5^ target cells. AKF9‐specific TCR‐T cells or CTL clone cells were incubated with or without the anti‐PD‐1 antibody nivolumab at a final concentration of 20 nM for 3 h before analysis. In some experiments, AKF9‐specific TCR‐T cells were activated using a Dynabeads CD3/CD28 T‐cell Expander (Thermo Fisher Scientific) for 72 h or 10 μM chloroquine diphosphate (Sigma‐Aldrich) for 48 h to upregulate PD‐1 expression.^19^, ^20^

### 2.10 Establishment of doxycycline (DOX)‐inducible HSF1 knockdown

The Tet‐pLKO‐puro plasmid (#21915) was purchased from Addgene (Cambridge, MA). Plasmid construction was performed according to the protocol provided by Addgene. Briefly, oligo sequences specific to HSF1 were identified from the MISSION® shRNA web site (Sigma‐Aldrich, www.sigmaaldrich.com), and five HSF1‐specific candidate oligos were designed. The following sequences were used in this study: Oligo‐A sense, 5′‐CCGGGCAGGTTGTTCATAGTCAGAACTCGAGTTCTGACTATGAACAACCTGCTTTTT‐3′ and Oligo‐A antisense, 5′‐AATTAAAAAGCAGGTTGTTCATAGTCAGAACTCGAGTTCTGACTATGAACAACCTGC‐3′; Oligo‐B sense, 5′‐CCGGGCCCAAGTACTTCAAGCACAACTCGAGTTGTGCTTGAAGTACTTGGGCTTTTT‐3′ and Oligo‐B antisense, 5′‐AATTAAAAAGCCCAAGTACTTCAAGCACAACTCGAGTTGTGCTTGAAGTACTTGGGC‐3′. The constructed plasmids were confirmed by sequencing. To establish stable transfectants, 293 T cells were co‐transfected with pLKO‐TetON plasmid, psPAX2 (#12260; Addgene), and pMD2.G (#12259; Addgene). After 2 days, the culture supernatant was obtained and used as a source of lentivirus. HSC2 cells were infected with lentivirus for 3 days, and then puromycin was added at 0.4 μg/ml to select the infected cells.

### 2.11 Establishment of HSC2 cells stably expressing the AKF9 neoantigen or HSF1‐S326A mutant

For establishing AKF9 neoantigen expressing HSC2 cells, DNA was introduced into HSC2 cells by a retrovirus‐mediated method.^21^ Briefly, the culture supernatant of PLAT‐A cells transduced with the pMXs‐based retrovirus vector containing myc‐epitope‐tagged AKF9 antigen was added to HSC2 cells in the presence of 8 μg/ml polybrene (Santa Cruz Biotechnology). For establishment of HSF1‐S326A mutant‐overexpressing HSC2 cells, pIRES‐puro3 (Takara, Kusatsu, Japan) plasmid carrying cDNA coding HSF1‐S326A mutant was transfected into HSC2 cells using Lipofectamine® 2000 Transfection Reagent (Invitrogen) according to manufacturer's protocol. cDNA of HSF1‐S326A mutant was obtained by PCR mutagenesis using mutation‐specific primers, and the sequence was confirmed by direct sequencing. Since after 3 days from transfection, the medium was replaced with fresh medium containing 0.4 μg/ml puromycin to select the infected cells.

### 2.12 Statistical analysis

The bar graphs in the figures present means ± standard deviation. Differences in variables were assessed using Student's *t*‐test. *p* < 0.05 was considered significant. The experiments were repeated at least three times and representative data are shown.

## 3 RESULTS

### 3.1 Transient CDDP exposure increases PD‐L1 expression on OSCC cells

**FIGURE 1:**
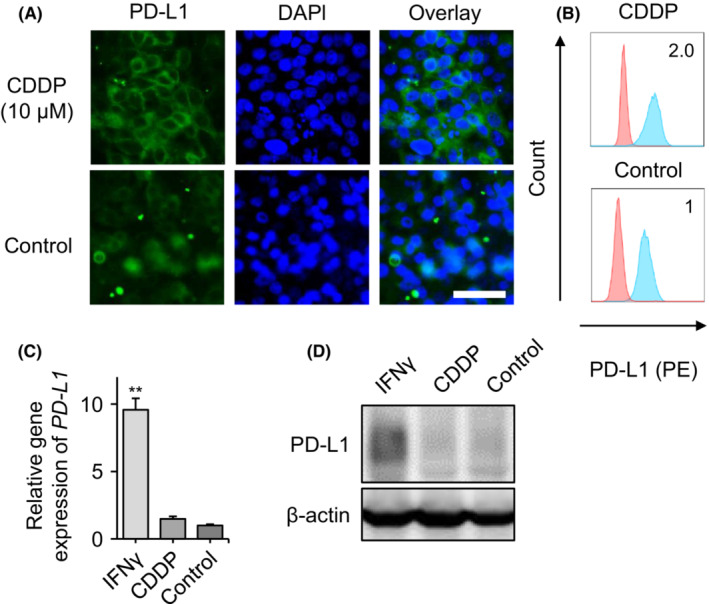
Expression of programmed death‐ligand 1 (PD‐L1) in HSC2 oral squamous cell carcinoma in response to cisplatin (CDDP). (A) Fluor‐labeled immunostaining of PD‐L1 in HSC2 cells with or without 10 nM CDDP stimulation for 24‐h. Nuclei were counterstained with 4′, 6‐diamidino‐2‐phenylindole. Bar = 50 μm. (B) Flow cytometry analysis of the cell surface expression of PD‐L1 on HSC2 cells with or without 10 nM CDDP stimulation for 24‐h. Numerical data indicate relative delta mean fluorescence intensity. (C) Transcriptional expression and (D) Western blot analysis of PD‐L1 in HSC2 cells in response to 24‐h stimulation with 5 ng/ml IFNγ or 10 nM CDDP. **p < 0.01.

The platinum‐based chemotherapeutic agent CDDP is one of the most commonly applied drugs for OSCC. Thus, we investigated whether CDDP affects PD‐L1 expression in OSCC cells using fluoro‐immunocytochemistry. CDDP (10 μM) increased the surface levels of PD‐L1 (Figure 1A). CDDP‐induced PD‐L1 overexpression was also confirmed with flow cytometry (Figure 1B). This result was confirmed in another OSCC cell line (HSC4 cells, Figure S1A). However, the expression of PD‐L2, another ligand for PD‐1, did not change in response to CDDP treatment (Figure S2).

We then investigated *PD‐L1* gene expression by quantitative real‐time PCR. Although IFNγ significantly increased *PD‐L1* gene expression, CDDP had a negligible effect on its expression (Figure 1C). In addition, the total protein level of PD‐L1 was not increased in response to CDDP stimulation, which was different from IFNγ stimulation (Figure 1D). These results were replicated in HSC4 cells (Figure S1B).

### 3.2 CDDP induces the activation of HSF1 and expression of HSPs

**FIGURE 2:**
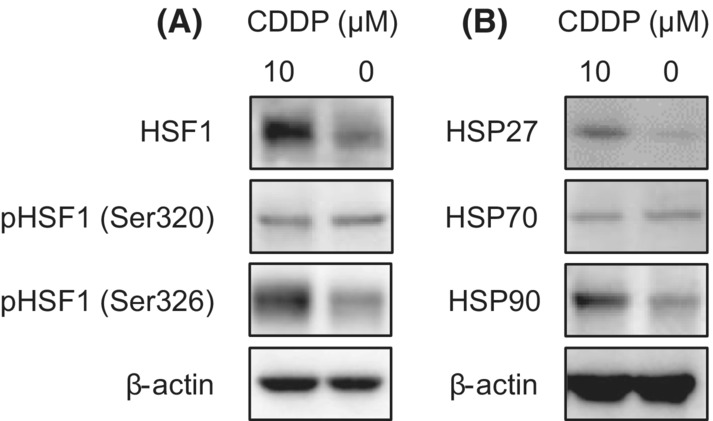
Expression of heat shock factor (HSF) 1 and heat shock proteins (HSPs). (A) Western blot analysis of total HSF1 and HSF1 phosphorylated at S320 or S326 in HSC2 cells after 12‐h stimulation with 10 nM CDDP. (B) Western blot analysis of HSP27, HSP70, and HSP90 in HSC2 cells after 24‐h stimulation with 10 nM CDDP.

The antitumor effect of CDDP is mediated by crosslinking the DNA double helix structure and consequent inhibition of DNA replication. In addition to this classic function, accumulating evidence shows that CDDP induces endoplasmic reticulum stress, eventually leading to the activation of the heat shock response.^22^, ^23^ Diverse HSPs have been implicated in the endoplasmic reticulum stress response.^24^ HSF1 is a master transcriptional regulator of a variety of HSPs.^25^ Thus, we hypothesized that the activation of HSF1 and HSPs would play a role in PD‐L1 surface expression. Then, we investigated the phosphorylation of HSF1 at representative serine residues associated with activation: serine 320 (S320) and serine 326 (S326).^26^ CDDP induced HSF1 phosphorylation at S326, while S320 phosphorylation was not altered (Figure 2A). Accordingly, we confirmed the increased expression of HSP27 and HSP90 in HSC2 cells in response to CDDP exposure, whereas HSP70 expression was not altered in the same setting (Figure 2B).

### 3.3 HSF1 knockdown modulates the cell surface expression of PD‐L1, but not its total protein level

**FIGURE 3:**
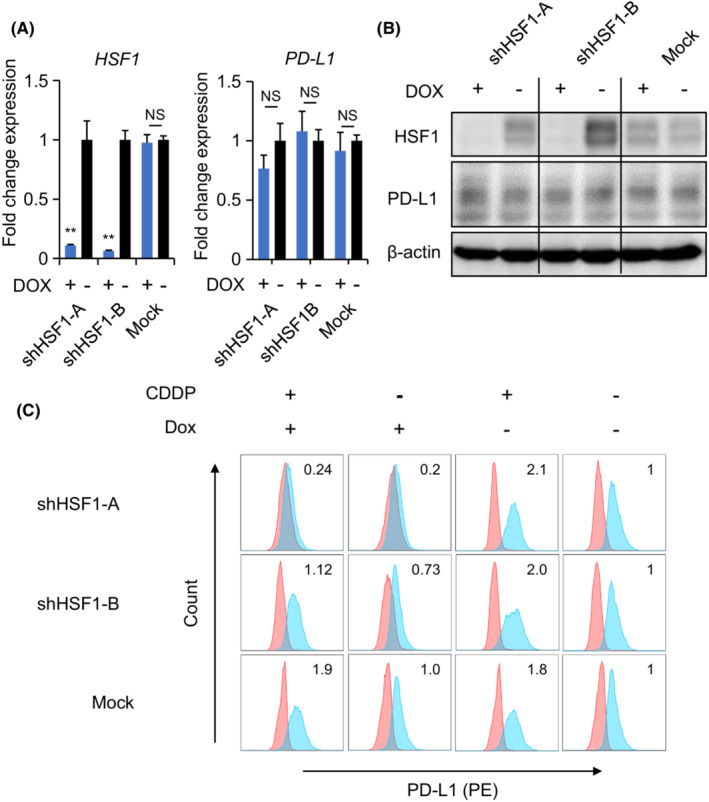
Cell surface PD‐L1 downregulation in the doxycycline (DOX)‐inducible HSF1‐suppressing HSC2 cell line. (A) Transcriptional expression and (B) western blot analysis of HSF1 and PD‐L1 in the DOX‐inducible HSF1‐suppressing HSC2 cell line. (C) Flow cytometry analysis of cell surface PD‐L1 expression on DOX‐inducible HSF1‐suppressing HSC2 cells with or without 10 nM CDDP stimulation for 24 h. Numerical data indicate relative delta mean fluorescence intensity. **p < 0.01.

To investigate the significance of HSF1 in PD‐L1 expression, we established DOX‐inducible HSF1 knockdown HSC2 cells using two different short‐hairpin RNA constructs. Although the transcriptional suppression of HSF1 was observed, *PD‐L1* gene expression was not altered significantly (Figure 3A). Furthermore, HSF1 knockdown did not alter PD‐L1 protein expression (Figure 3B). However, HSF1 suppression decreased the surface levels of PD‐L1. In addition, CDDP‐induced PD‐L1 overexpression on the cell surface was inhibited by HSF1 suppression (Figure 3C).

### 3.4 HSF1 S326A downregulates PD‐L1 expression

**FIGURE 4:**
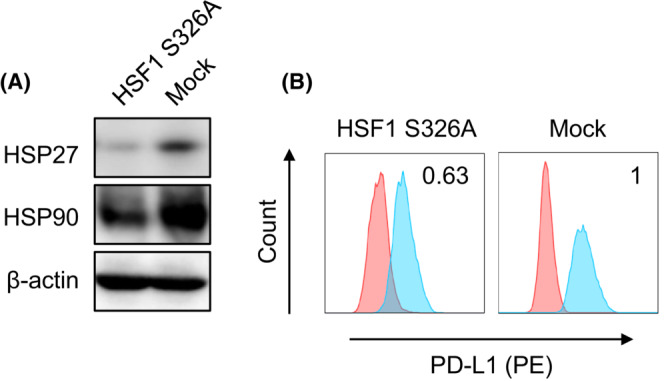
Decreased HSP27, HSP90, and cell surface PD‐L1 expression in HSC2 cells overexpressing the HSF1 S326A mutation. (A) Western blot analysis of HSP27 and HSP90 levels in HSC2 cells overexpressing the HSF1 S326A mutant. (B) Flow cytometry analysis of cell surface PD‐L1 expression on HSC2 cells overexpressing the HSF1 S326A mutant. Numerical data indicate relative delta mean fluorescence intensity.

We next investigated how HSF1 phosphorylation at S326 influenced PD‐L1 expression. HSC2 cells stably transformed with an HSF1‐S326A mutant, mimicking dephosphorylation of HSF1 at S326, were established as previously described.^27^ This HSF1‐S326A mutant‐overexpressing HSC2 cell line had attenuated HSP27 and HSP90 expression, consistent with the observation for CDDP exposure (Figure 4A). At the same time, PD‐L1 expression was decreased in S326A mutant HSC2 cells (Figure 4B).

### 3.5 HSP90 inhibition interferes with the induction of PD‐L1 by CDDP

**FIGURE 5:**
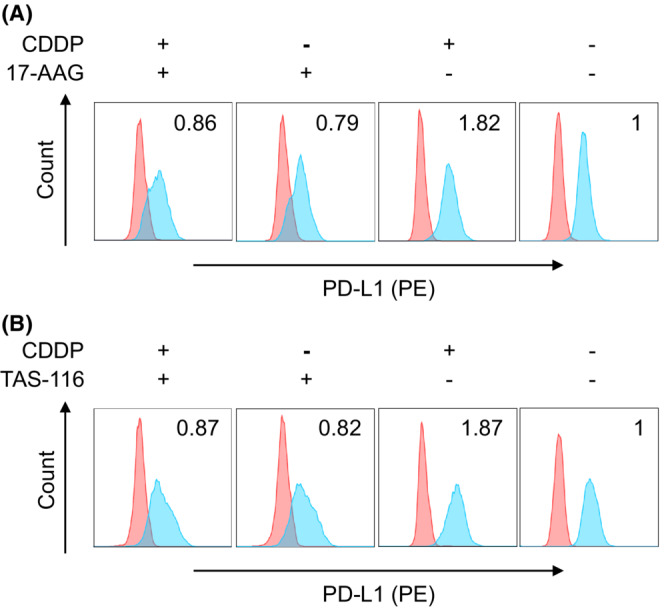
Cell surface PD‐L1 expression in response to HSP90 inhibition. Flow cytometry analysis of cell surface PD‐L1 expression on HSC2 cells treated with (A) 17‐AAG or (B) TAS‐116. HSC2 cells were treated with or without 1 μM of 17‐AAG or 3 μM TAS‐116 for 6 h and then incubated in culture medium with or without 10 μM CDDP for 24 h. Numerical data indicate relative delta mean fluorescence intensity.

We investigated whether HSP27 and/or HSP90 were involved in PD‐L1 expression. To clarify the involvement of HSP90 in PD‐L1 expression on HSC2 cells, we utilized two potent HSP90 inhibitors, 17‐AAG (tanespimycin) and TAS‐116 (pimitespib). Treatment with 1 μM of 17‐AAG (IC50: 5 nM) reduced PD‐L1 expression; moreover, 17‐AAG inhibited CDDP‐mediated PD‐L1 upregulation in HSC2 cells (Figure 5A). In addition, 3 μM TAS‐116 (IC50: <50 nM), also induced the same effects (Figure 5B). Nevertheless, the total protein level of PD‐L1 assessed by Western blotting was not altered (Figure S3A,B). In contrast, when HSP27 was downregulated using siRNA, CDDP‐mediated PD‐L1 upregulation was not inhibited (Figure S4A,B). Because HSPs bind to numerous client proteins, thereby aiding the stabilization or translocation of these proteins as molecular chaperones, we hypothesized that PD‐L1 is a client protein of HSP90 and consequently performed an immunoprecipitation experiment. Although HSP90 was detected by immunoblotting in HSC2 cell lysate immunoprecipitated using an anti‐PD‐L1 antibody, a specific band was not found in the rabbit gamma globulin control, indicating the direct binding of PD‐L1 and HSP90 (Figure S3C).

### 3.6 CDDP‐induced PD‐L1 expression dampens the antigen‐specific CTL response, which is antagonized by nivolumab‐mediated PD‐1 inhibition

**FIGURE 6:**
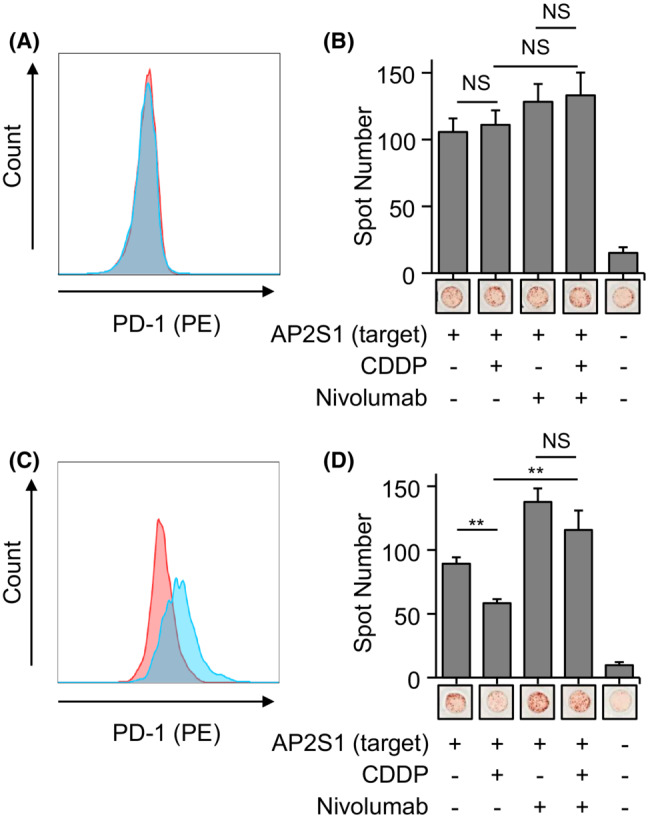
Functional relevance of CDDP‐induced PD‐L1 expression on HSC2 cells investigated by assessment of interferon (IFN)‐γ production activity by TCR engineered CD8 positive T cell processing AKF9 specific TCR (AKF9‐TCR‐T) cells. Flow cytometry analysis of PD‐1 expression on AKF9‐TCR‐T cells in the (A) absence or (C) presence of CD3/CD28 bead stimulation. (B), (D) IFNγ ELISpot assay investigating the activation of AKF9‐TCR‐T‐targeting HSC2 cells expressing the AKF9 neoantigen under the indicated conditions. (B) shows AKF9‐TCR‐T cells without CD3/CD28 bead stimulation. (D) indicates the condition of exhausted (i.e., PD‐1 expressing) AKF9‐TCR‐T cells with CD3/CD28 bead stimulation. **p < 0.01; NS, not significant.

We then investigated the functional significance of the increased surface levels of PD‐L1 in response to CDDP in HSC2 OSCC cells. Previously, AKF9, a highly antigenic neoantigen originating from the *AP2S1* c.258C > G gene mutation in HCT15 colon carcinoma cells, was identified in our laboratory.^13^ This neoantigen is presented by the HLA‐A24 allele. Using mutant AP2S1 as a model neoantigen, we established a mutant AP2S1‐overexpressing HSC2 cell line (AKF9‐HSC2). We have also established AKF9 peptide‐specific TCR‐T cells using a TCR derived from AKF9‐specific T cells as previously described.^14^ In the natural state, AKF9‐TCR‐T cells did not express PD‐1, a CTL exhaustion marker (Figure 6A). To investigate the antigen‐specific AKF9‐TCR‐T reaction, we assessed IFNγ production as a surrogate marker by an IFNγ‐ELISpot assay. There was no significant difference in IFNγ production in AKF9‐HSC2 cells between the presence and absence of the CDDP‐induced increase of PD‐L1 expression (Figure 6B). This was also confirmed in naturally isolated and expanded CTL clones from peripheral blood mononuclear cells targeting AKF9 (AKF9‐CTLs) (Figure S5A,B). In contrast, stimulation with CD3/CD28 beads mimicking the antigen–specific stimulation of the TCR increased PD‐1 expression in AKF9‐TCR‐T cells (Figure 6C). For naturally isolated and expanded CTLs targeting the AKF9 antigen, chloroquine instead of CD3/CD28 stimulation induced PD‐1 expression, as previously reported (Figure S5C).^20^ In this setting of PD‐1 expression on CTLs, the number of CTLs producing IFNγ in response to AKF9‐HSC2 cells was decreased when AKF9‐HSC2 cells had been exposed to CDDP (Figure 6D and Figure S5D). This inhibition of the antigen‐specific AKF9‐TCR‐T reaction was partly reversed by nivolumab, a clinically used anti‐PD‐1 antibody.

In this experiment, CDDP was removed from the culture medium before the assay; therefore, AKF9‐TCR‐T or AKF9‐CTL cells were not exposed to CDDP because after the removal of CDDP, increased PD‐L1 expression was maintained in AKF9‐HSC2 cells during AKF9‐TCR‐T or AKF9‐CTL exposure (Figure S6).

## 4 DISCUSSION

In this report, we demonstrated that CDDP increased the cell surface expression of PD‐L1 on HSC2 OSCC cells and was mediated by HSF1 and HSP90.

Although ICI monotherapy has been a successful approach for cancer immunotherapy, an increasing number of studies have shown the promising potential for combination therapy with ICIs and chemoradiotherapy.^9^ However, chemoradiotherapy is still considered to behave dualistically as a double‐edged sword in cancer immunotherapy. Cytotoxic chemotherapy can induce immunogenic cell death, which enhances the so‐called cancer immunity cycle, an essential process in clinically effective cancer immunotherapy.^28^ In addition, some cytotoxic chemotherapies can selectively eliminate immune‐suppressing cells such as regulatory T lymphocytes.^29^ However, cytotoxic chemotherapy can disturb anticancer immunity by suppressing immune reactions or inducing myelosuppression.

As another type of chemotherapeutic agent, CDDP also induces antitumor immunomodulation.^30^ CDDP can induce major histocompatibility complex (MHC) class I expression in tumor cells, the recruitment of CD8^+^ CTLs, or immunogenic death. According to our observations, CDDP, one of the oldest chemotherapeutic agents still in clinical use for OSCC, enhances the downregulation of the anticancer immune reaction by increasing PD‐L1 cell surface levels. In line with our current study, theoretically, the combination of CDDP and PD‐1 blockade would further promote anticancer efficiency. Indeed, in lung squamous cell carcinoma, an anti‐PD‐1 antibody (pembrolizumab) plus chemotherapy including platinum‐based compounds improves overall survival.^31^ This type of combination therapy has not been reported in OSCC; nevertheless, it seems to be a matter of time before this is assessed. The dose, timing, or route of administration should be investigated to optimize this approach so that the maximum clinical effect can be obtained. Our results help to provide a basic immunological insight into the reaction of OSCC to CDDP.

The present study revealed that the mRNA and total protein levels of PD‐L1 were not altered, although the cell surface levels of PD‐L1, which functions as an immune checkpoint molecule, were increased in response to CDDP. We hypothesize that a post‐transcriptional mechanism including molecular chaperoning underlies this process. The molecular chaperones CKLF‐like MARVEL transmembrane domain‐containing 4 and 6 (CMTM4 and CMTM6) are well‐known stabilizers of PD‐L1.^32^, ^33^ These proteins inhibit the lysosomal degradation of PD‐L1, thereby promoting the recycling and redistribution of functional PD‐L1 on the cell surface. In these studies, total PD‐L1 protein levels were also increased by CMTM stabilization of PD‐L1, implying the existence of a different mechanism for the post‐transcriptional regulation of PD‐L1. Indeed, CMTM4 and CMTM6 expression in response to CDDP was not altered (data not shown), leading us to focus on the functional significance of HSF1 and HSPs in the cell surface distribution of PD‐L1. In its role as a transcriptional activator, the phosphorylation status of HSF1 is considered to be important. Among a number of possible serine and threonine residues, phosphorylation at S320 and/or S326 of HSF1 is associated with transcriptional activation.^26^ CDDP stimulation of HSC2 cells increased S326 phosphorylation in HSF1. Of note, an S326A mutant mimicking the dephosphorylation of HSF1 at S326 decreased PD‐L1 expression in HSC2 cells. Therefore, the phosphorylation status of HSF1 at S326 would be, at least partly, involved in PD‐L1 expression. In our observations, PD‐L1 was not a transcriptional target of HSF1, which regulates numerous HSP family proteins including, HSP27, HSP90, and the HSP40/DNAJ family that consists of almost 50 proteins.^34^ Because the alteration of HSP27 and HSP90 expression was correlated with PD‐L1 levels in response to CDDP and overexpression of the S326A mutant, we consequently hypothesized that HSP27 or HSP90 played a role in PD‐L1 expression. We did not investigate every protein under the control of HSF1; nevertheless, our results indicate that HSP90 was involved in PD‐L1 expression. HSP90 is a pleiotropic protein that assists with protein sorting as well as proper folding.^35^ In this process, direct binding between HSP90 and PD‐L1 was confirmed, revealing PD‐L1 as a client protein of HSP90.

HSP expression is induced by endogenous or exogenous stress including heat shock, and HSPs physiologically correct the folding of misfolded proteins, thereby maintaining intracellular homeostasis. Various types of malignant tumors constitutively overexpress HSPs, which do not only refold misfolded proteins but also inhibit apoptosis.^36^, ^37^ Cancer cells are considered to be dependent on HSPs, thereby surviving in harsh environments. However, HSPs themselves are not characterized as oncogenic proteins but they promote the survival of cancer cells.^38^ An increasing number of reports have suggested that the inhibition of HSP90 with or without cytotoxic chemotherapeutics or molecular targeted agents would be a promising approach for effective cancer therapy.^39^ The present study revealed that the chemical inhibition of HSP90 downregulated PD‐L1 expression, suggesting the significance of HSP90 in cancer immunity. Importantly, HSP90 inhibition is reported to downregulate PD‐L1/PD‐L2 expression on antigen‐presenting cells.^40^ In the context of physiological immune tolerance, cellular stress‐induced PD‐L1 expression may play a role preventing excessive tissue damage as a feedback mechanism. Then, could the systemic administration of HSP90 inhibitors replace the use of anti‐PD‐1/PD‐L1 antibodies? Unfortunately, we cannot necessarily be optimistic for the answer to this question. HSP90 inhibition shows a dual action in cancer immunotherapy.^41^ Although HSP90 inhibition increases the expression of MHC class I molecules, it also reduces efficient peptide loading on MHC class I molecules in malignant cells.^42^, ^43^ In addition, HSP90 plays a cardinal role in cross‐presentation by antigen‐presenting cells.^44^ Consequently, HSP90 inhibition may attenuate efficient CTL activation in the priming phase. To further complicate the problem, we have to consider the effect of HSP90 inhibition on T cells, including CD8^+^ and CD4^+^ cells, which pivotal effector and essential helper cells in cancer immunotherapy, respectively. In one study, HSP90 inhibition enhanced CTL‐mediated cancer immunotherapy.^45^ In contrast, HSP90 inhibition ameliorated CD4^+^ T cell‐mediated graft versus host disease, a phenomenon on the other side of the coin to cancer immunotherapy.^46^ Due to its pleiotropic functions, it is important to perform further investigations of HSP90 inhibition from the viewpoint of dose, timing, or combination with other therapeutic agents.

In conclusion, we have shown that CDDP treatment enhanced PD‐L1 expression in OSCC cells. In this process, the HSF1‐HSP90 axis played an important role. Other than CDDP, many therapeutic interventions including non‐CDDP drugs or irradiation can induce cellular stress. Our results may provide valuable insight into therapy combining cytotoxic chemotherapeutics, molecular‐targeted agents, and immunotherapy. We believe that further investigation of the behavior of HSPs would provide a deeper understanding as well as novel therapeutic strategies for OSCC.

## AUTHOR CONTRIBUTIONS

**Takashi Sasaya:** Conceptualization (equal); data curation (equal); investigation (lead); writing – original draft (equal); writing – review and editing (equal). **Terufumi Kubo:** Conceptualization (lead); data curation (lead); formal analysis (lead); investigation (supporting); writing – original draft (lead); writing – review and editing (lead). **Kenji Murata:** Investigation (supporting). **Yuka Mizue:** Investigation (supporting). **Kenta Sasaki:** Investigation (supporting). **Junko Yanagawa:** Investigation (supporting). **Makoto Imagawa:** Investigation (supporting). **Hirotaka Kato:** Investigation (supporting). **Tomohide Tsukahara:** Conceptualization (supporting); data curation (supporting); formal analysis (supporting); funding acquisition (supporting). **Takayuki Kanaseki:** Conceptualization (supporting); data curation (supporting); formal analysis (supporting); funding acquisition (supporting). **Yasuaki Tamura:** Conceptualization (supporting); data curation (supporting); formal analysis (supporting). **Akihiro Miyazaki:** Supervision (equal). **Yoshihiko Hirohashi:** Conceptualization (equal); data curation (equal); formal analysis (equal); funding acquisition (supporting); validation (lead); writing – original draft (equal); writing – review and editing (equal). **Toshihiko Torigoe:** Conceptualization (supporting); data curation (supporting); formal analysis (supporting); funding acquisition (lead); project administration (lead); supervision (lead); validation (equal); writing – original draft (supporting); writing – review and editing (supporting).

## CONFLICT OF INTEREST

T. Torigoe has received research support from Ono Pharmaceutical, Dainippon Sumitomo Pharma, Medical & Biological Laboratories, Nippon Boehringer Ingelheim and Sapporo Clinical Laboratory. The rest of the authors declare that they have no relevant conflicts of interest for this work.

## ETHICS STATEMENT

Approval of the research protocol by an Institutional Reviewer Board.

## INFORMED CONSENT

N/A.

## REGISTRY AND THE REGISTRATION NO. OF THE STUDY/TRIAL

N/A.

## ANIMAL STUDIES

N/A.

## Supporting information


Data S1

## Data Availability

Data available within the article or its supplementary materials.
