# Supplementary figures and images for: Cisplatin‐induced HSF1‐HSP90 axis enhances the expression of functional PD‐L1 in oral squamous cell carcinoma
Source: Cancer Med. 2022 Oct 6;12(4):4605–15. doi: 10.1002/cam4.5310 (PMC9972142; doi:10.1002/cam4.5310)

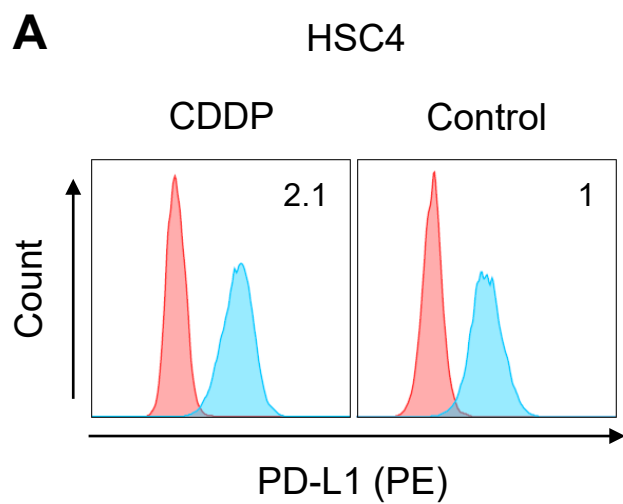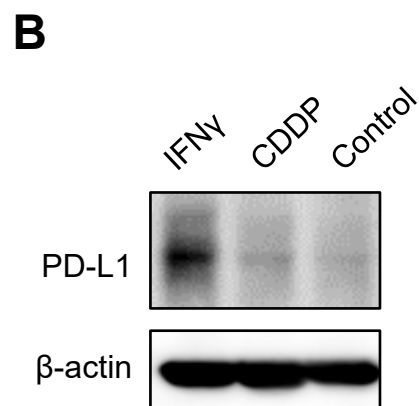

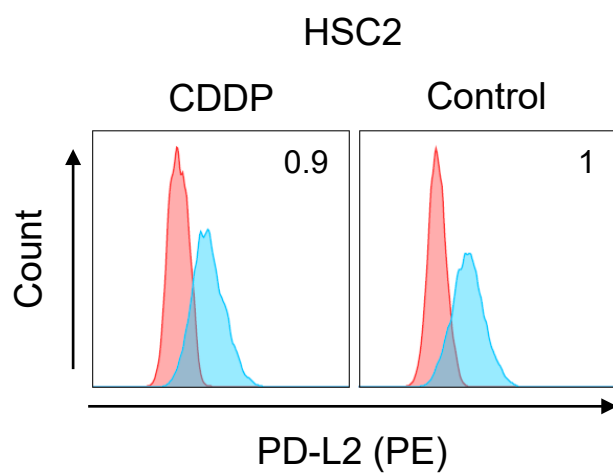

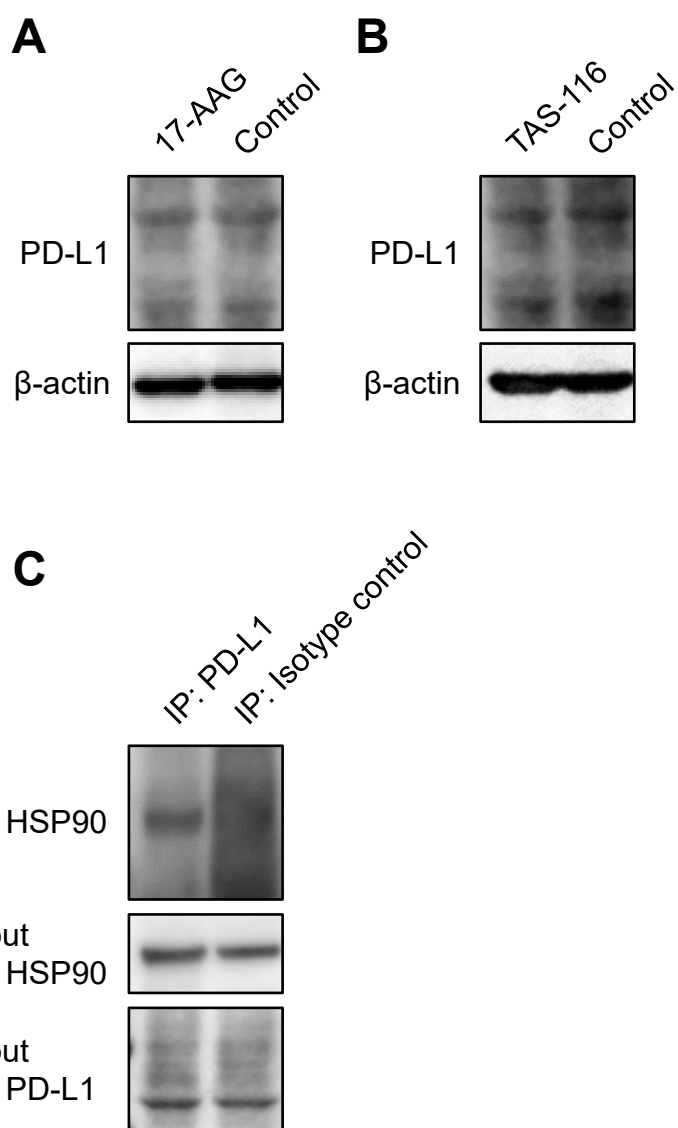

**A**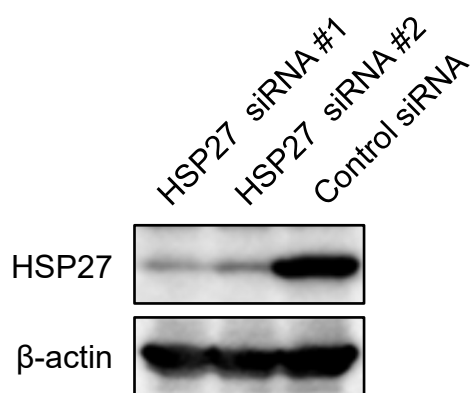**B**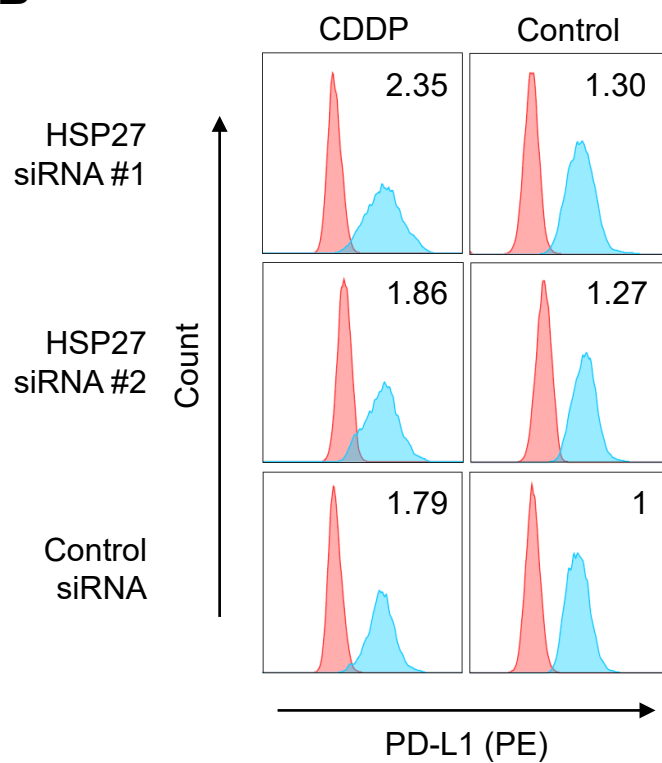

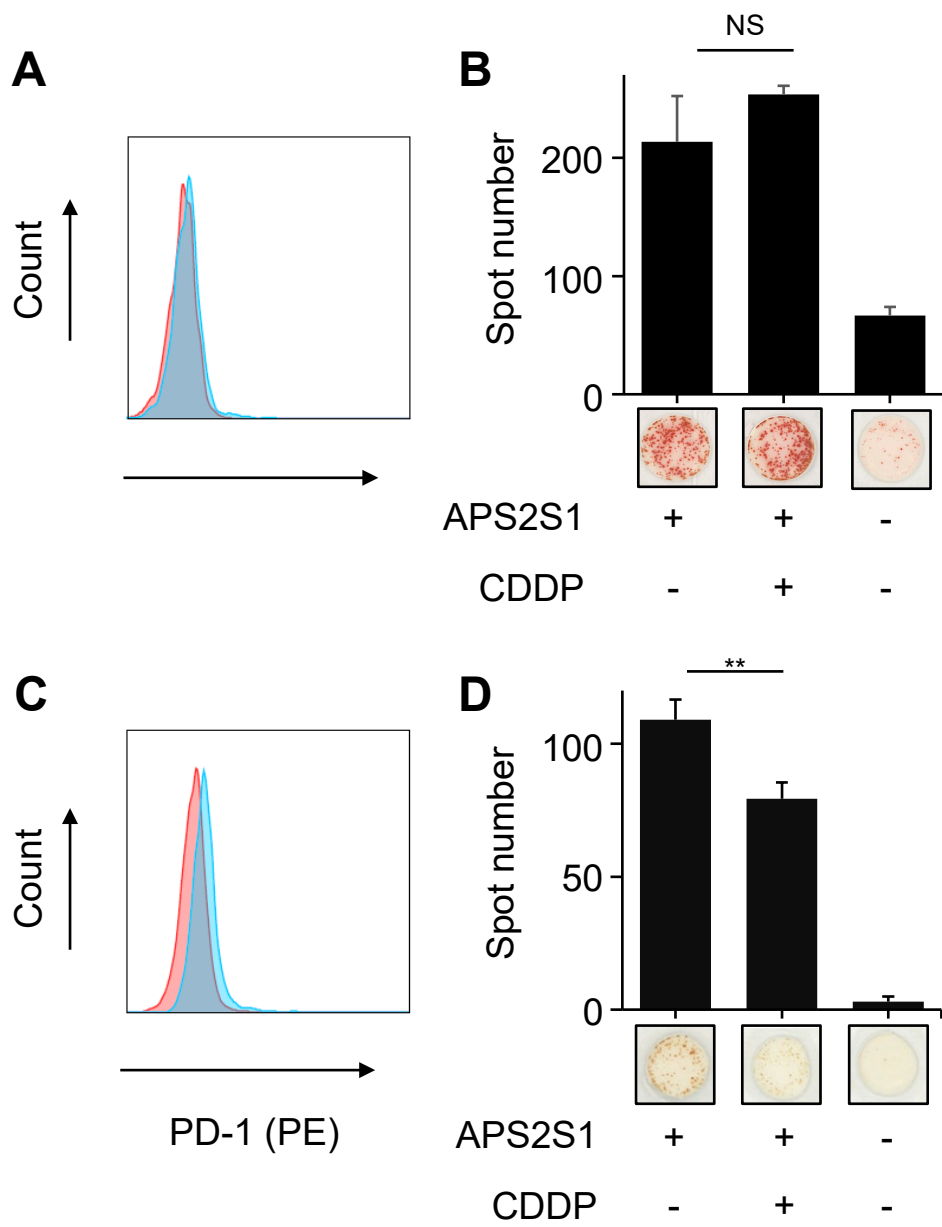

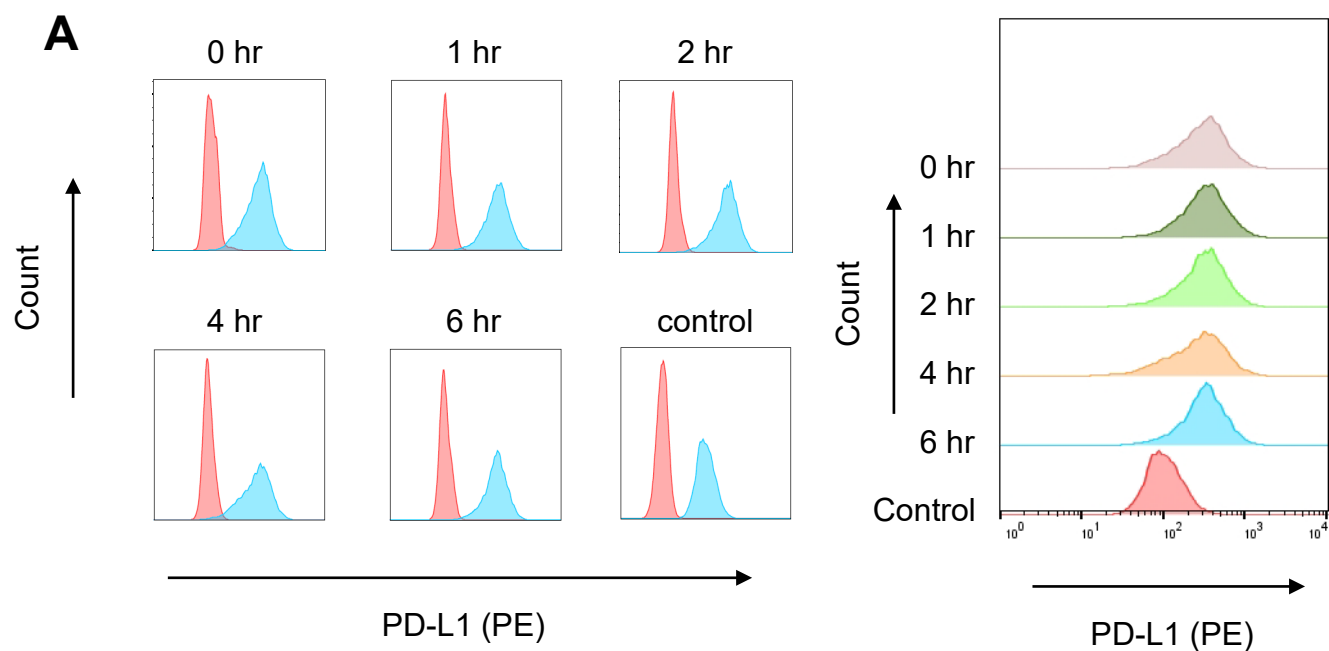

Supplement: Supplementary file 1 — Data S1 [file CAM4-12-4605-s001.pdf]
